# Composition and electronic structure of ${\rm SiO}_{\rm x}$/${\rm TiO}_{\rm y}$/Al passivating carrier selective contacts on n-type silicon solar cells

**DOI:** 10.1038/s41598-023-29831-2

**Published:** 2023-02-22

**Authors:** Christoph Flathmann, Tobias Meyer, Valeriya Titova, Jan Schmidt, Michael Seibt

**Affiliations:** 1 4th Institute of Physics– Solids and Nanostructures, University of Goettingen, 37077 Göttingen, Germany; 2 Institute of Materials Physics, University of Goettingen, 37077 Göttingen, Germany; 3 Institute for Solar Energy Research Hamelin (ISFH), 31860 Emmerthal, Germany; 4 Institute of Solid-State Physics, Leibniz University Hannover, 30167 Hannover, Germany

**Keywords:** Condensed-matter physics, Materials for devices, Materials for energy and catalysis, Nanoscale materials, Energy harvesting, Renewable energy, Applied physics

## Abstract

Carrier-selective and passivating SiO$_{\rm x}$/TiO$_{\rm y}$ heterocontacts are an attractive alternative to conventional contacts due to their high efficiency potentials combined with relatively simple processing schemes. It is widely accepted that post deposition annealing is necessary to obtain high photovoltaic efficiencies, especially for full area aluminum metallized contacts. Despite some previous high-level electron microscopy studies, the picture of atomic-scale processes underlying this improvement seems to be incomplete. In this work, we apply nanoscale electron microscopy techniques to macroscopically well-characterized solar cells with SiO$_{\rm x}$/TiO$_{\rm y}$/Al rear contacts on n-type silicon. Macroscopically, annealed solar cells show a tremendous decrease of series resistance and improved interface passivation. Analyzing the microscopic composition and electronic structure of the contacts, we find that partial intermixing of the SiO$_{\rm x}$ and TiO$_{\rm y}$ layers occurs due to annealing, leading to an apparent thickness reduction of the passivating SiO$_{\rm x}$. However, the electronic structure of the layers remains clearly distinct. Hence, we conclude that the key to obtain highly efficient SiO$_{\rm x}$/TiO$_{\rm y}$/Al contacts is to tailor the processing such that the excellent chemical interface passivation of a SiO$_{\rm x}$ layer is achieved for a layer thin enough to allow efficient tunneling through the layer. Furthermore, we discuss the impact of aluminum metallization on the above mentioned processes.

## Introduction

Carrier selective passivating contacts are of great interest for photovoltaic applications, as they potentially allow to improve solar cell efficiencies and to simplify production processes^1,2^. In contrast to conventional solar cells, no doping of the absorber material for creating a p–n-junction is needed when hole and electron selective contacts are combined for charge carrier separation^3^. Moreover, various selective contact materials also show high interface passivation^4^, reducing interface recombination losses and dispensing the need for heavily doped regions to tailor charge carrier conductivity. To make a good carrier selective contact, a material needs to have a large difference in electron and hole conductance, while the conductance for the selected carrier type should be as high as possible. When this property is combined with good interface passivation, it is possible to obtain high open circuit voltages (V$_{\rm oc}$) and high fill factors (FF)^5^.

Transition metal oxide contacts are reported as hole selective contacts^6–9^, while low work function insulators are known as electron selective contacts^10–12^. A particularly interesting contact material is titanium oxide (TiO$_{\rm y}$), which can be applied either as electron or as hole selective contact^13^. The electron selectivity is attributed to the asymmetric band offsets with respect to Si^14^. To increase conductivity and electron selectivity, oxygen deficient TiO$_{\rm y}$ (y < 2) is frequently employed^14–18^. The improvement is usually attributed to oxygen vacancy states, resulting in n-doping. Therefore, TiO$_{\rm y}$’s electron conductivity increases with increasing amount of oxygen vacancies^19^. In order to enhance the interface passivation, a few nm thick silicon oxide (SiO$_{\rm x}$) tunnel layer can be deposited between Si and TiO$_{\rm y}$^14,16^. In previous studies, differently processed SiO$_\text{2 }$ layers have been used, such as thermal oxides^14,20^, chemical oxides^21^ as well as native oxides with varying thickness^20^. Even TiO$_{\rm y}$ deposition on H-terminated silicon leads to the formation of a thin SiO$_\text{2 }$ interlayer upon annealing^22–24^ with beneficial effects on passivation.

Even though transmission electron microscopy (TEM) is capable of measuring structure and composition of such thin layers, the number of TEM studies on SiO$_{\rm x}$/TiO$_{\rm y}$ based electron selective contacts is limited. So far, Yang et al.^14^ and Ali et al.^16^ reported high resolution TEM (HRTEM) and energy filtered transmission electron microscopy measurements on SiO$_2$/TiO$_2$/Al contacts. From those, they were able to infer that the reduction of TiO$_2$ by Al is an essential step for obtaining low contact resistance. Furthermore, Dwivedi et al.^25^ and Mochizuki et al.^21^ performed HRTEM and scanning transmission electron microscopy (STEM) as well as electron energy loss spectroscopy (EELS) measurements on SiO$_{\rm x}$/TiO$_{\rm y}$ layers. Using these measurements, they could relate the improved interface passivation after annealing to the formation of Si, Ti and O containing layers. Despite those impressive insights gained by TEM, there are no comparable studies published for SiO$_{\rm x}$/TiO$_{\rm y}$/Al carrier selective contacts yet, making it difficult to draw a comprehensive conclusion about the composition-property relationship of such contacts.

The aim of this study is to elucidate how composition changes on a nanoscale affect the electronic properties of atomic layer deposition (ALD) grown, n-Si/SiO$_{\rm x}$/TiO$_{\rm y}$/Al carrier selective contacts with native SiO$_{\rm x}$ layers. As was previously shown^20,26–28^, solar cells with such contacts exhibit quite poor interface passivation and high series resistance in the as-deposited state. After a low temperature annealing step though, both properties improve considerably, leading to efficiencies of up to 20.3% for the batch of solar cells investigated here^26^ and 22.1% for the current record solar cells with SiO$_{\rm x}$/TiO$_{\rm y}$ based contacts^1^. However, the detailed reasons for these findings are not unequivocally clear yet. In particular, the importance of layer intermixing and how it affects the electronic structure of the contacts is not completely understood. In order to relate electronic properties to composition changes, analytical STEM methods are used to examine n-Si/SiO$_{\rm x}$/TiO$_{\rm y}$/Al solar cell rear contacts in this work. To clarify which properties improve the contacts’ efficiency, an as-deposited solar cell is compared to a cell annealed in ambient air for 30 min at 350 $^\circ$C.

## Results

To relate solar cell properties to structure and composition changes of the electron-selective contact stacks brought about by low-temperature annealing, solar cell characteristics are first described (“Solar cell characteristics” section). This is followed by a paragraph combining high angle annular darkfield (HAADF) STEM imaging with energy dispersive X-ray spectroscopy (EDX) (“Scanning transmission electron microscopy and energy dispersive X-ray spectroscopy” section). For a deeper insight into the chemical environment of the involved atomic species, i.e. silicon, oxygen, titanium and aluminum, spectrum images of the respective EELS core-loss edges, obtained along line scans across the rear contact, are presented (“Electron energy loss spectroscopy” section).

### Solar cell characteristics

**Figure 1:**
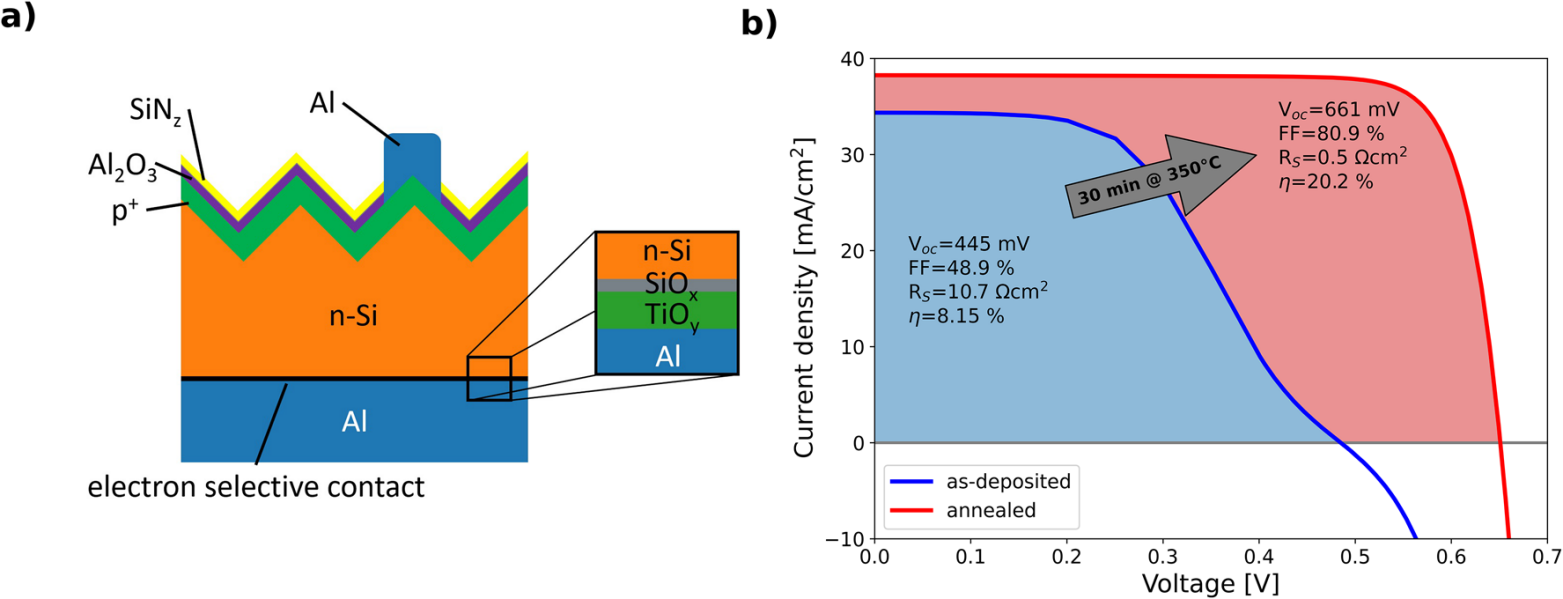
Characteristics of the investigated solar cells. (**a**) Schematic of the solar cells used for this study. The intended layer stack of the electron selective rear contact is shown enlarged in the black frame. (**b**) Presents the current-voltage curves of the as-deposited (blue) and annealed (red) solar cells, showing the strong improvement of solar cell efficiency with annealing.

A schematic of the investigated solar cells is presented in Fig. 1a. The fabrication process of the solar cells, including the annealing procedure, is specified in the “Methods” section. I–V measurements of an as-deposited and an annealed solar cell are shown in Fig. 1b. The I–V curves clearly reveal that the efficiency increases strongly due to the annealing step. V$_{\rm oc}$ increases from 485 mV in the as-deposited state to 651 mV in the annealed state, while the short circuit current (I$_{\rm sc}$) increases from 34.35 mA/cm$^2$ to 38.25 mA/cm$^2$ and the FF improves from 48.9 to 80.9%. The improvement of V$_{\rm oc}$ can be related to an improved interface passivation at the rear contact due to the annealing. Similarly, an increase of I$_{\rm sc}$ also indicates improved interface passivation. The increase of the FF can be related to an improved series resistance (R$_{\rm s}$), which drops from the initially very high value of 10.7 $\Omega$cm$^2$ to 0.5 $\Omega$cm$^2$ after annealing. This improvement in conductivity is further highlighted by the apparent S-shape of the as-deposited I–V curve, pointing towards a rather high, voltage dependent series resistance, which vanishes for the annealed curve. In total, an efficiency increase from 8.15 to 20.2% is observed, showing that a treatment at elevated temperatures and the corresponding reactions are essential for obtaining good contact properties of the examined solar cell contacts. For data on the passivation quality of the contacts, the reader is referred to Ref.^29^, where passivation data is extensively studied.

### Scanning transmission electron microscopy and energy dispersive X-ray spectroscopy

**Figure 2:**
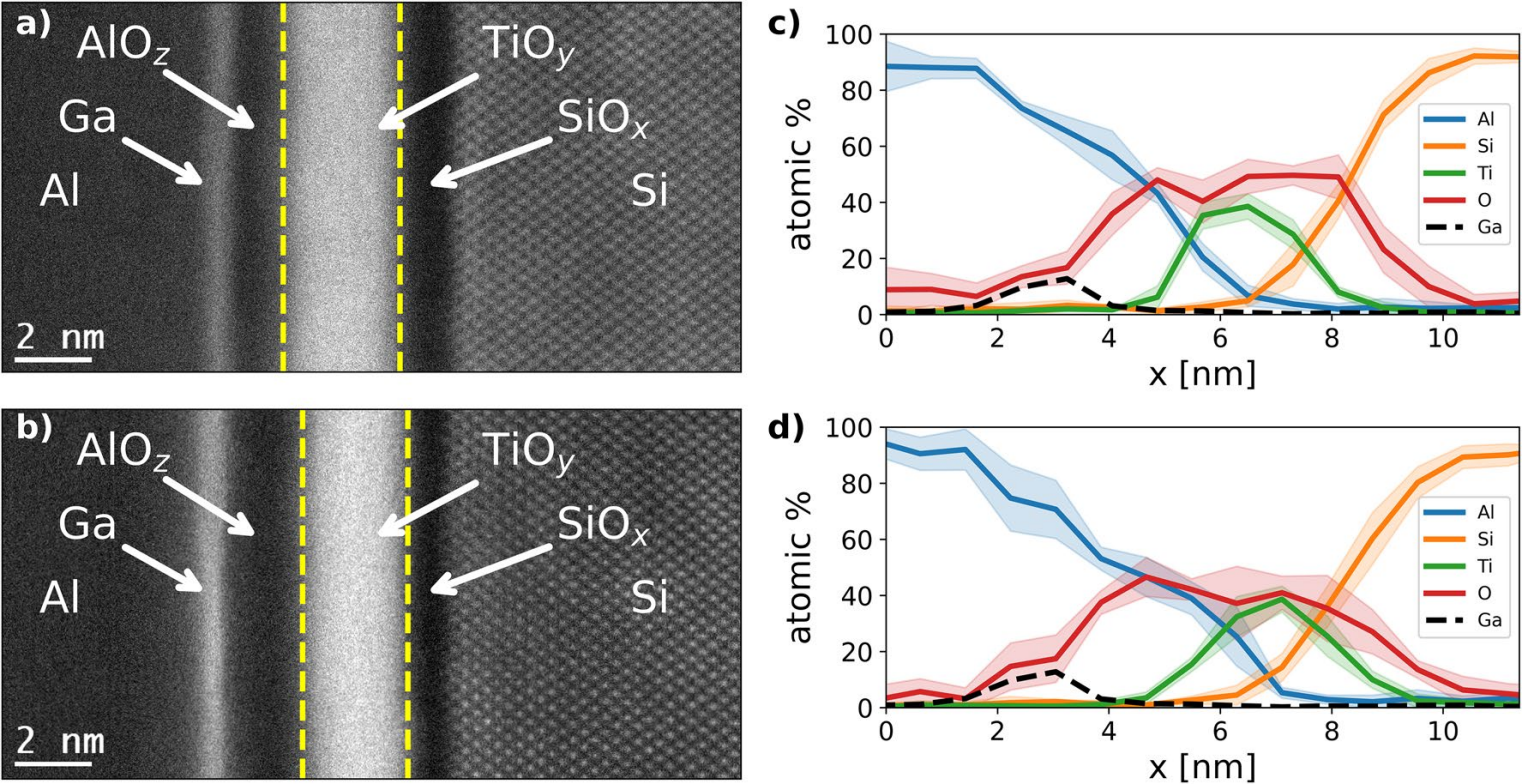
Rear contact layers and composition. (**a,b**) Show HAADF images of the as-depositedand the annealed solar cell rear contacts. The different layers of the contact stack are labeled, yellow dashed lines are added to illustrate the extend of the TiO$_{\rm y}$. (**c,d**) Depict the average of four EDX line scans over the contacts for as-deposited (**c**) and annealed (**d**) solar cells. The main constituents are depicted as solid lines, while Ga contamination is represented by a black, dashed line. The shaded areas represent plus-minus twice the standard deviation of the scans.

To determine the origin of the improved current-voltage characteristics, analytical TEM methods are carried out. High resolution HAADF STEM images of both specimens are presented in Fig. 2a,b. The typical dumbbell structure of Si in [110] zone axis orientation is visible in both cases on the right-hand side, with a sharp transition to the amorphous layers. No significant lateral inhomogeneities of the layers are observed over the entire width of the TEM lamellae. Layers in the amorphous part can be assigned straightforward due to the variations in HAADF intensity. The dark contrast next to the crystalline Si is SiO$_{\rm x}$, followed by a bright TiO$_{\rm y}$ layer. A second bright layer is observed further to the left. As can be seen from the EDX scans in Fig. 2c,d, this contrast is due to Ga contamination, probably as a result of differential milling at the interface of soft and hard layers during lamellae preparation. Hence, it is a helpful feature to visualize the aluminum oxide (AlO$_{\rm z}$) layer between the two bright layers, whereas the Al is located to the left of the Ga layer.

The most prominent feature, when comparing the as-deposited and the annealed sample in Fig. 2a,b, is an increase of the AlO$_{\rm z}$ layer thickness. Taking line profiles, averaged along the interface direction, and measuring the distance between inflection points, the extend of the layer can be estimated. For the as-deposited sample the thickness changes from 1.4 $\pm 0.1$ to 2.0 $\pm 0.1$ nm for the annealed sample. Here, the error due to lateral averaging and statistical fluctuations is estimated to be 0.1 nm. Moreover, the bright intensity of the TiO$_{\rm y}$ layer moves closer to the Si. This is substantiated by a decrease of TiO$_{\rm y}$–Si distance, measured as described above, from 1.4$\pm 0.1$ to to 1.0 $\pm 0.1$ nm.

In order to further quantify the findings, EDX scans are carried out. To obtain the results shown in Fig. 2c,d, four line scans over the layers are averaged for each sample. Quantifying the composition is achieved by integrating the intensity of the respective K lines and applying the Cliff–Lorimer method with k-factors specified for the employed EDX detector^30^. As the raw data shows negligible background, no background subtraction is needed prior to quantification. The error for the EDX measurements is obtained by calculating the standard deviation of the different scans.

The EDX results show that the Si distribution remains mostly unchanged by the annealing. Hence, the Si curve is used as reference to align the EDX measurements of the as-deposited and the annealed solar cell. Despite the rather large error in oxygen composition, the substoichiometry of the TiO$_{\rm y}$ layer is clearly recognizable for both contacts. Furthermore, the Ti curve shifts closer to the Si, being consistent with the SiO$_{\rm x}$ layer appearing thinner in the HAADF images. For the O curve, the opposite behaviour is observed. While the O concentration for the as-deposited sample is highest towards the Si side, with a secondary peak in the AlO$_{\rm z}$ layer, the O peak in the AlO$_{\rm z}$ layer increases for the annealed sample. This peak of the O curve coincides well with a shoulder of the Al curve, indicating advancing formation of an AlO$_{\rm z}$ layer. The observations confirm that the as-deposited solar cell already contains an AlO$_{\rm z}$ layer. However, a redistribution of O towards the Al side is observed for the annealed sample.

### Electron energy loss spectroscopy

In this section, EELS data is presented in order to gain additional insight into the chemical environment of involved atoms, which is encoded in the energy loss near edge structure (ELNES). Subsequently, we analyse the different ELNES data in two steps: “Phenomenological analysis” section identifies the chemical environment of involved atoms on a phenomenological basis mainly referring to energy positions of core-loss edges. “Data clustering” section then uses results of data clustering, explicating the effects of low-temperature annealing in detail.

#### Phenomenological analysis

**Figure 3:**
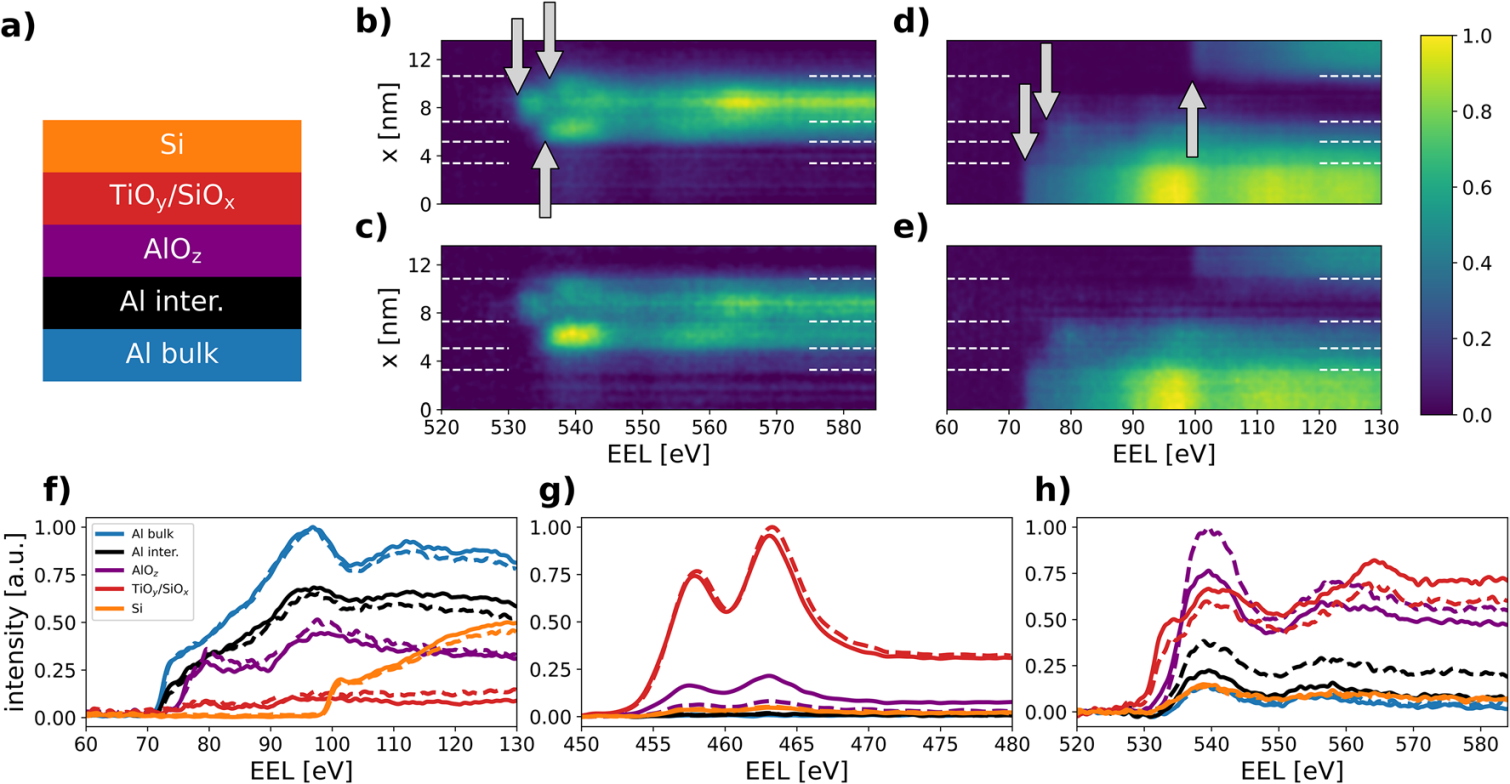
EELS results obtained from the rear contacts. (**a**) Depicts a schematic of the layers obtained by cluster analysis. (**b–e**) Show spectrum images over the contact region for (**b**) the O K-edge and (**d**) the Al/Si L-edge of the as deposited sample and (**c,e**) displaying the respective edges for the annealed sample. The edge onset energies are marked with arrows in (**b,d**). Interfaces of the layers given in (**a**) are indicated by white dashed lines in (**b–e**). The mean EEL spectra of these layers are presented in (**f–h**), where solid and dashed lines refer to the as-deposited and annealed state, respectively, and colors are in accordance with (**a**). (**f**) Shows the Al L$_{2,3}$/Si L$_{2,3}$ energy range, (**g**) shows the range around the Ti L$_{2,3}$ edge and (**h**) shows the range around the O K-edge.

The L$_{2,3}$-edges of Al (at about 72 eV) and Si (at about 99 eV), as well as the Ti L$_{2,3}$-edge (at about 455 eV) and the oxygen K-edge (at about 535 eV) are recorded simultaneously during EELS scans over the rear contacts. To separate the edge signals from the background, power law fits are subtracted from the raw data. By processing the data as described in Supplementary Information Sect. I, profiles of the EELS intensities for the aforementioned elements can be obtained along the scan direction. The resulting profiles are summarized in Fig. S1. They fully support observations from HAADF and EDX, in particular the increased oxygen intensity from the AlO$_{\rm z}$ and the shift of Ti intensity towards the Si as a result of annealing. Moreover, the Si EELS profiles do not change due to annealing, which means that they again serve as a reference to align the EELS scans of the as-deposited and the annealed sample.

As the O K-edge onset is determined by transitions from an oxygen 1s state to unoccupied valence states, its onset and near edge fine structure is related to bonding conditions^31,32^. Thus, it should be possible to distinguish the O containing layers based on their edge onset energy. For stoichiometric compounds onset energies of 535 eV for Al$_2$O$_3$^33^, 531 eV for TiO$_2$^34^ and 536 eV for SiO$_2$^32^ are found.

Figure 3b,c show the spectrally resolved O K-edge for the EELS line scans described above. In order to compare the as-deposited sample b and the annealed sample c, both data sets are normalized to their total intensity by integration over the position and energy dimension, and scaled to the range 0 to 1. Despite the previously observed strong intermixing of the layers, even the annealed sample shows a structure of three clearly distinguishable, O containing layers. The O K-edge onset energies are marked for each layer, showing that the O bonding conditions in those layers are comparable to Al$_2$O$_3$, TiO$_2$ and SiO$_2$. This not only justifies the labeling of the layers but also highlights the importance of tailoring the properties of those layers.

#### Data clustering

Further insight can be gained from the EELS data performing a cluster analysis ^35^. The goal of such procedures is to identify EEL spectra within the data sets, which are significantly distinct from each other. In the optimal case, the identified ’clusters’ correspond to different spatial regions of the measured line scans. For this purpose, K-Means clustering^36^ is carried out with five clusters, in an energy loss range from 60 eV to 130 eV, on both, as-deposited and annealed, data sets simultaneously. The data sets are shown in Fig. 3d,e, where the Al L$_{2,3}$-edge and the Si L$_{2,3}$-edge are marked with arrows for the Al, AlO$_{\rm z}$ and Si parts. A detailed description and discussion of the cluster analysis is given in Supplementary Information Sect. II. It should be pointed out that none of the clusters is specific to either the as-deposited or annealed sample. Hence, the annealing procedure modifies the intermixing and spatial distribution rather than the chemical environment of constituents. As all obtained clusters contain spatially contiguous scan positions, the EELS scans can be partitioned accordingly into five regions. These regions are visualized in Fig. 3; in (a) a schematic of the regions is shown, while the borders of the regions are indicated as white dashed lines in (b–e). Likewise, the clustered regions are indicated for the EELS data of the Ti L$_{2,3}$-edge in Supplementary Fig. S2.

The cluster borders, derived from the Al L$_{2,3}$/Si L$_{2,3}$ EELS data in Fig. 3d,e, fit remarkably well to the changes visible in the O K-edge intensity in Fig. 3b,c, providing evidence that the clustering procedure partitions the scan positions into physically meaningful subsets. Based on the knowledge about the samples’ composition, labels are assigned to the clusters as indicated in Fig. 3a. Comparing the spatial extent of the AlO$_{\rm z}$ cluster for the as-deposited and the annealed solar cell, an increase in thickness from 1.7 ± 0.1 to 2.2 ± 0.1 nm is found, matching very well with the values found by HAADF.

Moreover, the partitioning obtained by clustering can be used to calculate spatially averaged spectra for different energy loss ranges, i.e. the Al L$_{2,3}$/Si L$_{2,3}$ range, the Ti L$_{2,3}$ range and the O K range, thus enabling to correlate spectral information from different energy loss ranges in similar spatial regions. Mean EEL spectra are shown in Fig. 3f–h for each cluster, where solid lines indicate spectra from the as-deposited solar cell and dashed lines indicate spectra from the annealed solar cell. Fig. 3f shows spectra for the Al L$_{2,3}$/Si L$_{2,3}$ range, whereas (g) and (h) show spectra around the T L$_{2,3}$ and the O K-edge. In the Al L$_{2,3}$/Si L$_{2,3}$ range, as-deposited and annealed spectra look very similar indicating that the chemical environment of Al and Si is unchanged. The onset energy of the AlO$_{\rm z}$ spectra is significantly shifted to higher energy losses compared to the Al spectra, showing that the AlO$_{\rm z}$ cluster can be distinguished from the other Al containing regions.

For the Ti L$_{2,3}$ spectra, considerable intensities are only found for the TiO$_{\rm y}$/SiO$_{\rm x}$ and the AlO$_{\rm z}$ clusters, thus demonstrating that Ti is present in the AlO$_{\rm z}$ region. Furthermore, it is visible that the intensity of the mean Ti L$_{2,3}$-edge spectra in the AlO$_{\rm z}$ cluster decreases strongly after annealing. For the O-K edge, no changes are observed for the Al and Si clusters, While for the TiO$_{\rm y}$/SiO$_{\rm x}$ cluster, the O signal decreases due to annealing, proving a reduction, whereas further oxidation of the AlO$_z$ and the Al interface regions is manifested by the increased O signal of those spectra.

## Discussion

Assessment of the solar cells by means of their I–V curves shows that a substantial improvement in solar cell quality is achieved by an annealing step in ambient air. As was determined from the I–V characteristics, the most important improvements occur in terms of an enhanced interface passivation and a reduced series resistance. In the following, we shall discuss the presented TEM imaging and spectroscopy results, mainly focusing on these two aspects.

**Figure 4:**
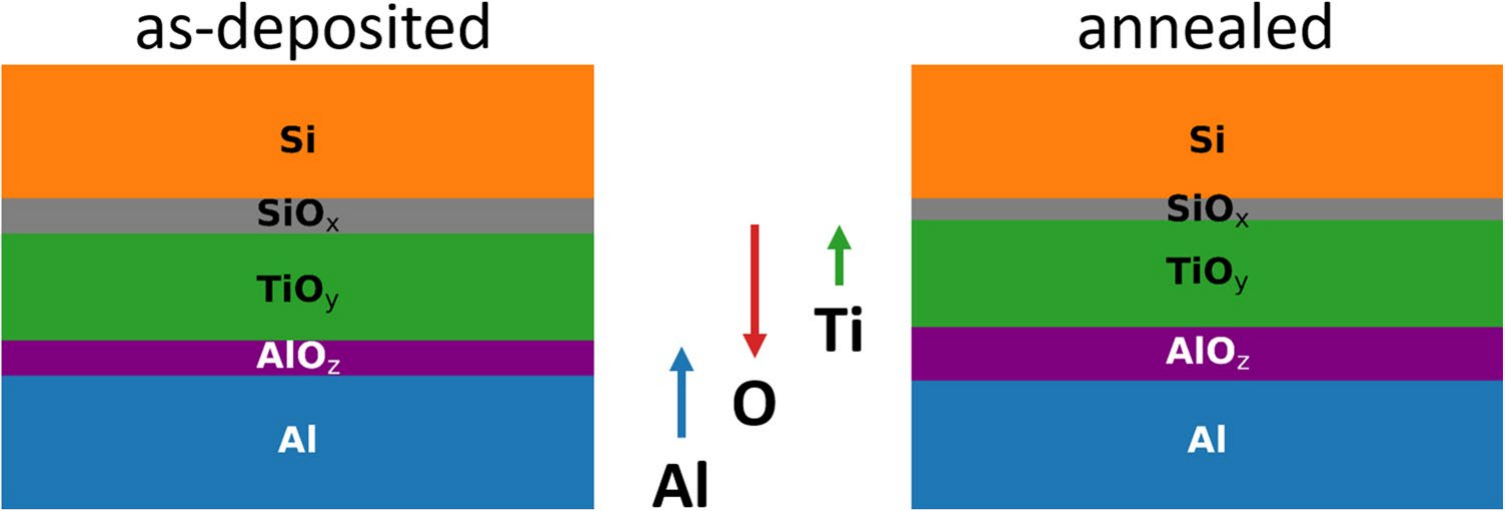
Schematic summary of observed layers (thicknesses are not to scale) and diffusion fluxes (arrows). ‘Si’ and ‘Al’ denote the silicon substrate and the aluminum rear contact, respectively. ‘SiO$_{\rm x}$’ is the passivating silicon oxide, which contains Ti. ‘TiO$_{\rm y}$’ denotes the titanium oxide layer moving towards the substrates as a result of titanium and oxygen diffusion; moreover, stronger oxygen deficiency is observed after annealing. ‘AlO$_{\rm z}$’ denotes the aluminum oxide layer being already present in the as-deposited sample; annealing leads to further oxidation and partial removal of titanium. Finally, ‘Al/AlO$_{\rm z}$’ denotes the interface between aluminum and aluminum oxide; according to EELS data it can be viewed as a superposition of the two adjacent phases.

Figure 4 graphically summarizes the observed layers and atomic transport occurring during 350 $^\circ$C annealing. HAADF and EELS spectrum imaging consistently show three different oxygen-containing layers for as-deposited and annealed solar cells. In particular, precise assessment of the O K-edge allows assignment of those layers as AlO$_{\rm z}$, TiO$_{\rm y}$ and SiO$_{\rm x}$ indicating that the AlO$_{\rm z}$ is already present in the as-deposited state and no new phases appear due to the annealing treatment. This important result is further corroborated by the cluster analysis of the EELS data, which finds identical clusters without and with annealing indicating composition changes rather than formation of new phases. The composition changes, as consistently observed by EDX and EELS, mainly comprise of oxygen redistribution towards the Al layer and Ti diffusion towards Si. As a result, the AlO$_{\rm z}$ layer thickness increases and intermixing of Ti and SiO$_{\rm x}$ occurs.

It has been convincingly shown, that the interface passivation of SiO$_{\rm x}$/TiO$_{\rm y}$ stacks mainly originates from chemical passivation^21^. The strong chemical passivation of near-stoichiometric SiO$_{\rm x}$ on crystalline Si originates from decreased interface defect density and the highly asymmetric capturing cross sections for electrons and holes at SiO$_{\rm x}$/Si interfaces^37,38^. In previous studies of TiO$_{\rm y}$ based contacts (see Refs.^21,25,39^), the formation of Ti-O-Si bonds is associated with improved interface passivation. This view is further supported by our observations, which in addition provide evidence that the intermixing is accompanied by a slightly reduced oxygen content at the SiO$_{\rm x}$/TiO$_{\rm y}$ interface (Figs. 2, 3h) and also Fig. S1 in the SI). Bearing in mind, that TiO$_2$ tends to oxidize silicon^22,23^ this observation provides a first clue to the importance of aluminum as a (strongly) reducing agent in the layer stack. The latter has also been related to hydrogen release^14^ during annealing and its beneficial effect for passivating interface defect states, an interpretation debated in terms of hydrogen effusion and nuclear reaction analysis^24^. Clearly, our experiments cannot contribute to this aspect since they are not sensitive to hydrogen. From our ELNES results, however, it can be concluded that the electronic structure of the SiO$_{\rm x}$ layer close to the Si interface is very similar to that of SiO$_2$ (see e.g. Ref.^32^) suggesting that the enhanced chemical passivation after annealing can mainly be related to the SiO$_{\rm x}$ layer observed by TEM techniques.

The resistance of the contact is determined by the resistance of the individual layers and their interfaces, it is reasonable to assume the total resistance is dominated by the contribution with the highest resistance. It is quite straightforward to conclude that the AlO$_{\rm z}$ layers do not significantly contribute the overall resistance, as the series resistance of the annealed solar cell is much smaller, even though the AlO$_{\rm z}$ layer is much thicker. As the AlO$_{\rm z}$ is present in the as-deposited state, it has to be formed during the deposition process. Therefore, it is likely non stoichiometric and defect rich. This presumption is supported by the EDX data showing even for the annealed state a composition far from Al$_2$O$_3$.

For TiO$_2$ based selective contacts it is known that reduction by the contact metal is crucial to decrease the resistance of the TiO$_2$ layer^14^. The sub-stoichiometric TiO$_{\rm y}$ layers presented in this study, however, appear to be highly oxygen deficient already in the as-deposited state. Therefore, it is unlikely that the huge decrease of the series resistance is solely caused by increasing the oxygen deficiency of the TiO$_{\rm y}$ layer. Indeed, recent studies^20,29^ on samples with virtually identical selective contact processing conditions to ours showed that the contact resistance of SiO$_{\rm x}$/TiO$_{\rm y}$ contacts is much higher compared to TiO$_{\rm y}$ ones and almost independent of TiO$_{\rm y}$ thickness in the as-deposited state, while for the annealed samples the contact resistances are almost identical and a clear dependence on TiO$_{\rm y}$ thickness is observed. This identifies the TiO$_y$ layer as limiting the contact resistance in the annealed sample, but excludes it for the as-deposited state. Thus, the SiO$_{\rm x}$ layer is limiting the conduction of the contact stack in the as-deposited state. This interpretation is strongly corroborated by our observation of decreased SiO$_{\rm x}$ layer thickness for annealed solar cells, as the tunnel current through the layer increases in this case. For the final thickness of the SiO$_{\rm x}$ layer of 1.0 nm a significant tunnel current is expected^32^. Moreover, an initially large contribution of the tunnel barrier to the series resistance is in agreement with the observed S-shape of the as-deposited I–V curve, since the tunnel resistance is voltage dependent. The vanishing of the S-shape for the annealed solar cell thus implies that the contribution of the tunnel barrier to the series resistance decreases strongly.

Notwithstanding the aforementioned, we can not exclude further processes from contributing to improved conductance. A recent simulation study for TOPCon solar cells showed that very low pinhole fractions, covering areal fractions of only a few tenth of a percent, can contribute significantly to the total current flow over an oxide layer^40^. In particular, when individual pinholes are small (a few nm diameter), such pinholes could not be observed in TEM. However, since the investigated SiO$_{\rm x}$ is very thin and the annealing temperatures are relatively low it appears reasonable that in the presented work tunneling is still the dominant mechanism^41^. Another effect could be the formation of titanium silicide at the TiO$_{\rm y}$/SiO$_{\rm x}$ interface. Even though we did not observe evidence for silicide formation in the EELS data, a positive contribution of silicides to the conductance can not be excluded, as silicide formation should only cause slight changes of the ELNES.

In order to complete this section, let us briefly comment on the role of the Al metallization. For this purpose, it is worth to consider the defect disorder of TiO$_2$ and its dependence on oxygen partial pressure^42^. Briefly, for oxidizing conditions (high oxygen partial pressure), dominant point defects are titanium vacancies (V$_{\rm Ti}^\text {2-}$) acting as acceptors, whereas for reducing conditions (small oxygen partial pressure) oxygen vacancies V$_{\rm O}^\text {2+}$ are dominant with smaller contributions from interstital titanium (Ti$_{\rm i}^\text {3+}$ and Ti$_{\rm i}^\text {4+}$) all acting as donors. Hence, reducing conditions will not only establish the desired n-type TiO$_2$, but also increase the number of highly mobile interstitial Ti atoms supporting the observed intermixing at the SiO$_{\rm x}$/TiO$_{\rm y}$ interface beneficial for the contact properties. There is ample evidence that the main effect of Al is to provide (strongly) reducing conditions during annealing. As discussed above, this not only establishes sufficient electron conductivity of the TiO$_y$ carrier selective layer, but also ensures strong intermixing at the SiO$_{\rm x}$/TiO$_{\rm y}$ interface and the critical reduction of the SiO$_x$ tunnel oxide thickness. Hence, any contact metal with an oxygen affinity comparable to that of aluminum (e.g. zirconium) is a potential candidate as a suitable material. We note, however, that in addition to the oxygen chemistry the oxide has to be sufficiently conductive for not increasing the contact resistance. Finally, we would like to mention that the final thickness of the AlO$_{\rm z}$ depends on the amount of available oxygen. As the contact stack is covered with a thick (1 $\upmu$m), full area Al layer during annealing, the only oxygen sources for AlO$_{\rm z}$ layer growth are the TiO$_{\rm y}$ and SiO$_{\rm x}$ layers. Thus, the reactions will adjust themselves to a certain extent, resulting in a rather robust process.

## Conclusion

We presented a detailed study on the impact of annealing on n-Si/SiO$_{\rm x}$/TiO$_{\rm y}$/Al carrier selective passivating contacts. Comparing macroscopic solar cell measurements with composition and electronic properties determined by TEM measurements, we were able to determine the most important adjustments leading to improved photovoltaic performance of the annealed cells. As the main improvements we identified a large decrease of the solar cell’s series resistance and enhanced interface passivation. Analyzing the electronic structure of the oxygen containing layers, we observed that for both, the as deposited and the annealed contact stack, a layer with an electronic structure close to that of SiO$_2$ exists. Thus, we conclude that the improved interface passivation is due to the excellent chemical passivation of that layer, which gets activated by the annealing procedure. Regarding the drop of the series resistance, our observations suggest that this is mainly caused by a decreased thickness of the SiO$_{\rm x}$ layer in the annealed state. The observed thickness after annealing of 1.0 nm is in a range were electrons can effectively tunnel through the layer and thus the resistance is no longer dominated by the SiO$_{\rm x}$ layer. Hence, our results show the importance of tailoring the SiO$_{\rm x}$ layer such that the excellent surface passivation of a SiO$_2$ layer is combined with sufficiently high tunnel currents. The detailed investigation of the composition further gives insight into chemical reactions taking place. This insight can help to optimize the fabrication procedure such that the full potential of sub-stoichiometric TiO$_{\rm y}$ carrier selective contacts can be reached.

## Methods

### Fabrication

To prepare the solar cells, an (100)-oriented phosphorous-doped Czochralski silicon wafer with a resistivity of 1.5 $\Omega$cm and an initial thickness of 300 $\upmu$m is utilized. After a standard RCA clean, a $\sim$ 200 nm thick SiO$_2$ protection layer is thermally grown on both wafer surfaces at a temperature of 1050 $^\circ$C in a quartz tube furnace (TS-81004, Tempress Systems). After the oxidation, 2 $\times$ 2 cm$^2$ windows are opened by laser ablation. The laser-ablated side defines the front side. Next, the front surface is random pyramid textured in KOH/wetting agent solution heated to a temperature of 80 $^\circ$C. The texturing reduces the final wafer thickness to a value of $\sim$ 290 $\upmu$m.

After texturing, the wafers receive another RCA clean and are subsequently boron-diffused. A p$^+$ emitter with a sheet resistance of $\sim$ 100 $\Omega$/sq. is formed on the front side in a quartz-tube furnace at 940 $^\circ$C in a BBr$_3$/O$_2$ atmosphere (TS-81004, Tempress Systems). Next, the borosilicate glass and the SiO$_2$ protection layers are removed in hydrofluoric acid. Subsequently, the originally 6 inch wafers are laser cut into 2.49 $\times$ 2.49 cm$^2$ samples for the subsequent cell fabrication. After laser cutting, all samples receive another RCA cleaning. As a next step, a 12 $\upmu$m thick Al grid (finger pitch: 1 mm, finger width: 20 $\mu$m) is evaporated through a Ni shadow mask as front side contact (BAK 550, Oerlikon Blazers AG). Subsequently, the front side is passivated with a 10 nm thick Al$_2$O$_3$ layer by plasma assisted atomic layer deposition (FlexAL, Oxford Instruments). Afterwards, a 52 nm thick SiN$_{\rm x}$ (refractive index n=1.9) layer is deposited at 300 $^\circ$C in a microwave remote plasma-enhanced chemical vapour deposition reactor (Plasmalab 80+ reactor, Oxford Instruments) onto the Al$_2$O$_3$ layer.

After the complete processing of the front side, the samples are stored in ambient environment for 3 months in order to grow an ultrathin native SiO$_{\rm x}$ on the entire cell rear. Past storage, the thickness of the natively grown SiO$_{\rm x}$ amounts to $\sim$ 1.3 nm. Subsequently, a 3 nm thick TiO$_{\rm y}$ layer is deposited on the rear using thermal ALD in a FlexAL reactor (Oxford Instruments) at a temperature of 230 $^\circ$C. Tetrakis(dimethylamino)titanium, H$_2$O and N$_2$ are used as titanium precursor, oxidant and purge gases, respectively. Finally, the entire rear surface is metallized with 1 $\upmu$m aluminum. For the Al metallization, e-beam evaporation is applied. A schematic of the solar cell is depicted as inset of Fig. 1.

To compare as-deposited and annealed solar cells, two parallel processed cells are used. While one cell is left in the state described above, the other cell is subsequently annealed in ambient environment, using the optimized annealing conditions of 30 min at 350 $^\circ$C^26^.

### Characterization

Current-voltage characteristics of the respective samples are measured on 4 cm$^2$ cells under 1 sun illumination employing a commercial LOANA System I-V Tester. The R$_{\rm s}$ values are extracted from the light I-V and I$_{\rm sc}$ - V$_{\rm oc}$ measurements under the illumination at one sun. For the as-deposited state, the measurements uncertainty is quite large, but R$_{\rm s}$ can still be clearly identified.

Cross section TEM lamellae are prepared from the rear contacts of the solar cells described in the previous section, using a standard focused ion beam lift out technique. Lamellae are then attached to copper grids and milled with a Ga ion beam down to thicknesses well below 100 nm. The ion acceleration voltage is gradually decreased from 30 kV during the first milling step, to 5 kV during the final milling step. Note that the Ga contamination, mentioned in “Scanning transmission electron microscopy and energy dispersive X-ray spectroscopy” section, developed during this step as a result of differential milling.

For TEM measurements an image corrected FEI Titan80-300, operated at 300 kV, is used. EDX scans are performed with an Oxford Instruments X-Max 80 mm$^2$ detector, capturing an energy range from 0 to 10 keV divided into 1024 channels. EELS is acquired employing a Gatan Quantum965 ER with an acceptance semi-angle of 39 mrad. It is operated in dual EELS mode, covering energy ranges from 50.0 to 254.8 eV and from 380.0 to 584.8 eV while using 2048 energy channels with an energy resolution of 0.1 eV per channel. Analysis of EDX and EELS data is performed with the python library HyperSpy^43^ as well as custom written python scripts. The displayed EELS data is smoothed using Savitzky–Golay filtering.

## Supplementary Information

Supplementary Information.

## Data Availability

The datasets generated during and/or analysed during the current study are available from the corresponding author on reasonable request.
