# Supplementary material for: Composition and electronic structure of SiOx/TiOy/Al passivating carrier selective contacts on n-type silicon solar cells
Source: Sci Rep. 2023 Feb 22;13:3124. doi: 10.1038/s41598-023-29831-2 (PMC9946942; doi:10.1038/s41598-023-29831-2)
Supplement: Supplementary file 1 — Supplementary Information. [file 41598_2023_29831_MOESM1_ESM.pdf]

Supplementary Information

Composition and electronic structure of $SiO_x/TiO_y/Al$ passivating carrier selective contacts on n-type silicon solar cells

Christoph Flathmann[1], Tobias Meyer[1,2], Valeriya Titova[3,4], Jan Schmidt[3,4] and Michael Seibt[1]

[1] 4th Institute of Physics– Solids and Nanostructures, University of Goettingen, 37077 Göttingen, Germany

[2] Institute of Materials Physics, University of Goettingen, 37077 Göttingen, Germany

[3] Institute for Solar Energy Research Hamelin (ISFH), 31860 Emmerthal, Germany

[4] Institute of Solid-State Physics, Leibniz University Hannover, 30167 Hannover, Germany

e-mail: mseibt@gwdg.de

I EELS profiles

Fig. S1 shows the EELS profiles for Si, Al, Ti and O. The data is extracted from the same background corrected EELS scans as shown in the main text. Al and Si EELS profiles are obtained by subtracting mean signals from pre- and post-edge energy ranges. The remaining mean background intensity is obtained in an energy range right in front of the respective EELS edge and is subtracted from the mean intensity obtained in an energy range right after the corresponding EELS edge. For obtaining the Ti and O EELS profiles, the mean intensity from a post-edge energy loss range is used directly. The obtained profiles are aligned using the Si curves as reference and are normalized by their integrated intensities.

The profiles show good agreement with the concentrations obtained from EDX and with the findings from HAADF images. For the O profile, shown in Fig. S1 d), a clear increase of intensity on the Al side is visible after annealing. Simultaneously, the O K-edge intensity on the Si side decreases and a pronounced double peak structure is observed. The annealed Al profile shows development of a plateau region at the position where the O profile peaks. This is probably related to formation of AlO_z with a certain stoichiometry. For the Ti profile, a shift towards the Si side is found for the annealed sample. Thus, indicating that Si and Ti intermix more strongly due to the annealing step. Neither for the as-deposited solar cell, nor for the annealed solar cell the peaks of the Ti profiles appear at the positions of the O profile peaks.

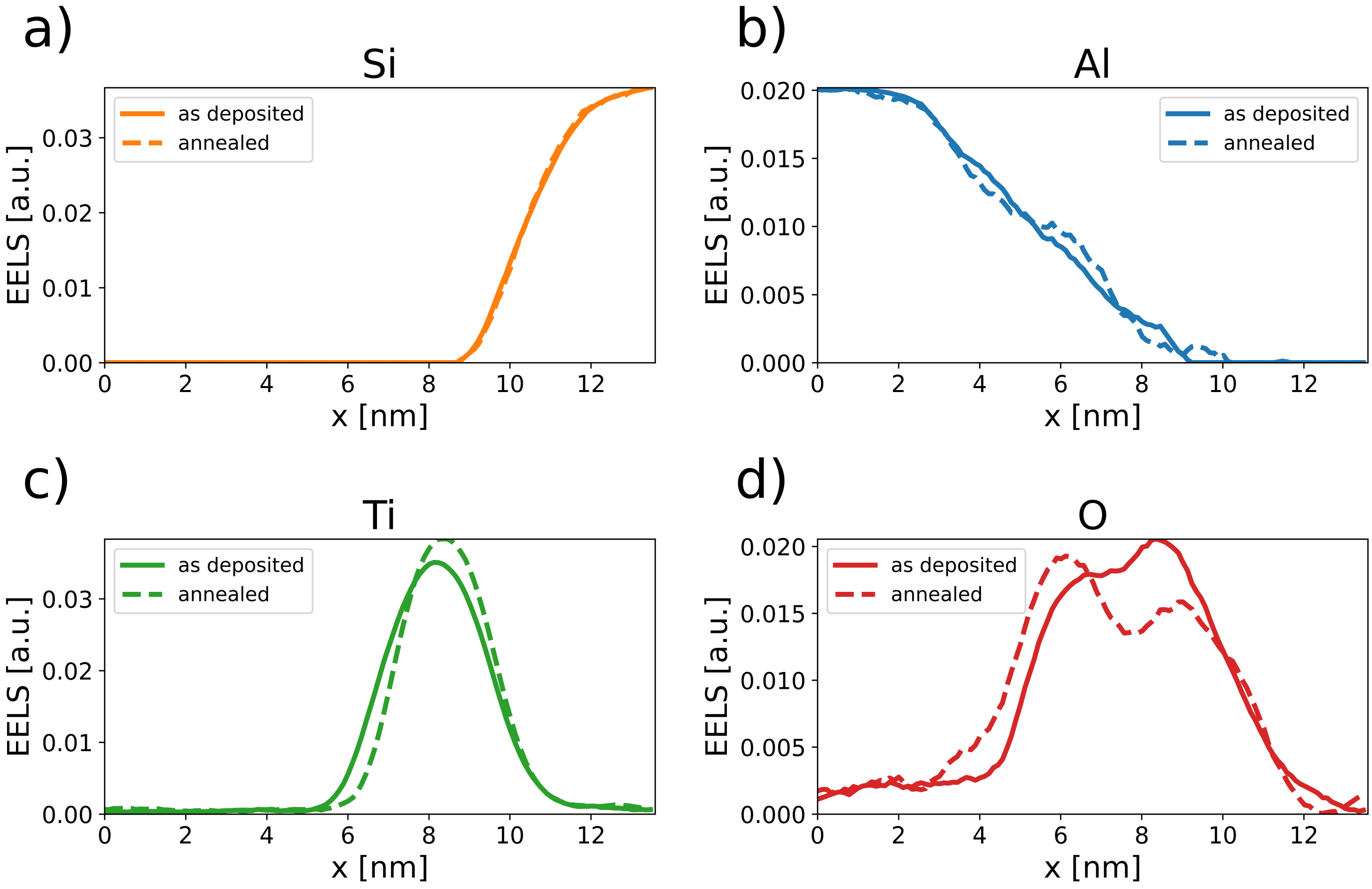

Fig. S1. Normalized EELS profiles, scanned across the contacts of the solar cells. a)-d) show the results for different elements, with the element being indicated in each panel. Curves for the as-deposited cell are represented by solid lines, while curves for the annealed cell are depicted as dashed lines. The Si curve is used as a reference to align as-deposited and annealed profiles.

II EELS data clustering

To prepare the EELS data for cluster analysis, the background corrected low loss range EEL spectra of the as-deposited and the annealed solar cell are cropped to the energy loss range 60-130 eV. Included in this energy range are the Al $L_{2,3}$-edge and the Si $L_{2,3}$-edge, where the former edge shows clearly different onset energies for Al and AlO_z. Subsequently, the cropped data sets are normalized with their intensity integrated over the spatial and the energy dimension. Moreover, the scans are shifted in the spatial dimension, so that the Si profiles shown in Fig. S1 are aligned. This procedure results in 122 aligned spectra for each EELS scan. In order to check weather annealing leads to qualitative changes of the spectra, the as-deposited scan and the annealed scan are concatenated along the spatial dimension. Hence, appearance or disappearance of spectral features after annealing would results in clusters being exclusively found in the annealed or the as-dposited spectra. Thus, the final data set consists of 244 spectra, hereafter referred to as samples, taken at 122 scan positions with each spectrum containing 700 energy channels with intensities values, hereafter referred to as features.

For clustering the data a K-Means approach is chosen. This approach aims to minimize the following expression:

$$\min \sum_{i=1}^{K} \sum_{\boldsymbol{x}_j \in C_i} \|\boldsymbol{x}_j - \boldsymbol{\mu}_i\|^2 \tag{1}$$

Where K is the number of clusters, $\boldsymbol{x}_j$ is the j-th sample, C_i is the set of samples being assigned to cluster i and $\boldsymbol{\mu}_i$ is the centroid of C_i. $\boldsymbol{x}_j$ and $\boldsymbol{\mu}_i$ are vectors having as many dimensions as the data set has features. To carry out the K-Means cluster analysis, the algorithm implemented in the python library scikit-learn is used[44]. In brief, for initialising the centroids, K samples are randomly chosen from the data set. After that, every sample is assigned to the cluster with the closest centroid. Followed by redetermining the centroids by calculating the mean of all samples assigned to the respective cluster. The last two steps are iterated until convergence is reached. The cluster algorithm is run with different numbers of cluster to find the optimum value for K. Values of $K = \{3, 4, 5, 6\}$ are tested. Based on reproducibility of clustering results and comparison of clusters to Ti $L_{2,3}$-edge and O K-edge spectra, 5 centroids are used to cluster the EELS data. It should be mentioned that, despite random starting conditions, K-Means clustering with $K = 5$ always finds the exact same clusters.

Borders between the five clusters, each consisting of a set of spatially contiguous samples, are overlaid as white dashed lines to the spectrum images shown in main text Fig. 3 b)-e). Very good agreement with the visual changes of the spectra is found, even though the EDX profiles showed gradual changes of the concentration between layers. Moreover, the cluster borders appear to be well aligned with features in the O K-edge and the Ti $L_{2,3}$-edge data shown in main text Fig. 3 b) and c) and Fig. S2. Nevertheless, these gradual concentration changes could be the reason for distinction between bulk Al and interface Al in the cluster analysis. However, it is also possible that this distinction is the result of changes of the composition and Al bonding properties close to the interface[45] or due to the previously mentioned Ga contamination during lamellae preparation in this region. Regardless of the reason for finding an Al interface cluster, the AlO_z seems to be determined reliably. This is supported by the increased thickness of the AlO_z cluster after annealing, being in good agreement with the increase found by HAADF. For the TiO_y and SiO_x layers a joint TiO_y/SiO_x cluster is found.

Nevertheless, the clusters represent physically meaningful entities. Hence, the mean EEL spectra of the clusters, presented in main text Fig. 3 f)-h), can be used to draw conclusions about sample properties. The differences in O K-edge intensity in Fig. 3 h) can be interpreted as a measure for how much O is on average in the corresponding region. For the Al and the Si clusters only very small O K-edge intensities are observed, likely being the result of oxidation of the lamellae surfaces. For the other clusters, significant O K-edge intensities are found. The increased intensity for the Al interface and the AlO_z spectra after annealing indicate oxidation of these regions, while the decreased intensity in the TiO_y/SiO_x cluster demonstrates reduction of this part of the contact. Considerable intensities for the mean Ti $L_{2,3}$-edge spectra, shown in main text Fig. 3 g), are only found for the TiO_y/SiO_x cluster and the AlO_z cluster. Hence, it can be concluded that Ti is incorporated in the region corresponding to the AlO_z cluster.The decrease of the Ti $L_{2,3}$-edge intensity in the AlO_z cluster after annealing is consistent with the observed Ti diffusion towards the Si.

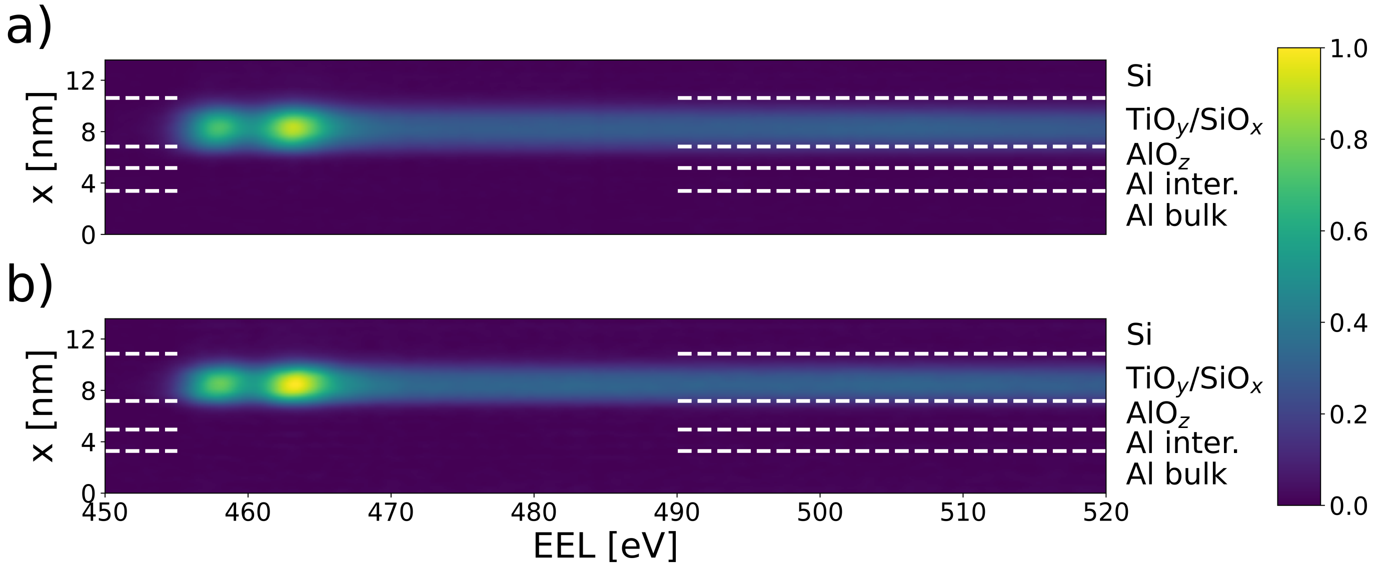

Fig. S2. a) normalized EELS spectrum image over the contact of the as-deposited solar cell, showing the Ti $L_{2,3}$-edge. b) normalized EELS spectrum image over the contact of the annealed solar cell, showing the Ti $L_{2,3}$-edge. Clusters are labeled and cluster borders are marked with white dashed lines.

References

[44] Pedregosa, F. *et al.* Scikit-learn: Machine Learning in Python. *Journal of Machine Learning Research* **12**, 2825–2830 (2011).

[45] Bouchet, D. & Colliex, C. Experimental study of ELNES at grain boundaries in alumina: intergranular radiation damage effects on Al-L23 and OK edges. *Ultramicroscopy* **96**, 139–152 (2003). https://doi.org/10.1016/S0304-3991(02)00437-0.
